# Supplementary figures and images for: Variations of thermophysical properties and heat transfer performance of nanoparticle-enhanced ionic liquids
Source: R Soc Open Sci. 2019 Apr 24;6(4):182040. doi: 10.1098/rsos.182040 (PMC6502364; doi:10.1098/rsos.182040)

**Velocity inlet    Pressure inlet**

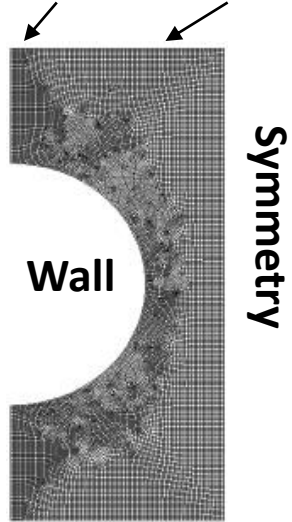

**Pressure out**

Supplement: Figure 3 [file rsos182040supp1.pdf]
